# Heterogeneity of recreationists in a park and protected area

**DOI:** 10.1371/journal.pone.0268303

**Published:** 2022-05-11

**Authors:** Olivia A. DaRugna, Mark A. Kaemingk, Christopher J. Chizinski, Kevin L. Pope

**Affiliations:** 1 Nebraska Cooperative Fish and Wildlife Research Unit, and School of Natural Resources, University of Nebraska-Lincoln, Lincoln, Nebraska, United States of America; 2 Department of Biology, University of North Dakota, Grand Forks, North Dakota, United States of America; 3 School of Natural Resources, University of Nebraska-Lincoln, Lincoln, Nebraska, United States of America; 4 U.S. Geological Survey—Nebraska Cooperative Fish and Wildlife Research Unit, and School of Natural Resources, University of Nebraska-Lincoln, Lincoln, Nebraska, United States of America; University of Regina, CANADA

## Abstract

Limited information and resources have caused many parks and protected areas (PPAs) to functionally manage recreationists as a single homogeneous group, despite potential negative social and ecological consequences. We aimed to evaluate the homogeneity of recreationists at the Valentine National Wildlife Refuge (NWR) by 1) quantifying frequencies of consumptive (i.e., hunting), intermediate-consumptive (i.e., fishing), and non-consumptive recreational-activity groups (e.g., wildlife viewing), and 2) evaluating sociodemographic differences among these groups. We used onsite surveys to determine that Valentine NWR supports heterogeneous groups of recreationists. The intermediate-consumptive group was most frequent (77% of all parties). All three recreational-activity groups varied in party size, distance traveled, household income, population type (urban or rural residence), and vehicle type (two-wheel or four-wheel drive). Tracking and accounting for diverse recreationists will equip managers with the ability to sustain recreational activities while also preserving ecological systems.

## Introduction

Parks and protected areas (PPAs) represent important social-ecological systems that serve dual purposes: 1) to preserve and manage ecological systems and 2) to provide wildlife-compatible recreational opportunities and ecotourism [1]. To effectively achieve these dual purposes, managers must account for both ecological and recreational diversities on these shared lands [1]. Many PPAs suffer from a lack of social and recreation information due to limited resources and difficulty of gathering and tracking this information [2]. Many PPAs allow multiple recreational activities, but few PPAs have quantified the types and frequencies of these activities, leading to functionally managing recreationists as a single homogeneous group. Managing for a homogenous recreational-activity group may have worked in the past, but this strategy will likely be unsuccessful in dealing with record-high levels of visitation [3]. Increased visitation can lead to social conflicts among diverse groups [4] and to various ecological impacts from different recreating groups [5].

Previous studies have used social media information and novel techniques to identify and track nonconsumptive recreational activities [6–9]; these methods could also be used to understand and manage consumptive recreational activities. Many PPAs allow for both consumptive and non-consumptive recreational activities [3, 10], but it is unclear whether these two groups should be managed as a homogenous group. These two recreational activities are expected to attract different sets of recreationists with varying characteristics [11]. Consumptive recreationists permanently extract (i.e., harvest) organisms from the environment; in contrast, non-consumptive recreationists do not intend to remove or permanently affect organisms [11].

Herein, we assessed whether it is appropriate to functionally manage recreationists as a single homogeneous group by 1) quantifying frequencies of understudied consumptive, intermediate-consumptive, and non-consumptive groups, and 2) evaluating differences in sociodemographic attributes among these three recreational-activity groups. We addressed these objectives using the Valentine National Wildlife Refuge (NWR), which like many PPAs permits a wide range of recreational activities. Recreational activities can be categorized into consumptive (hunting), intermediate-consumptive (fishing; anglers can be catch-and-release or harvest oriented), or non-consumptive (e.g., wildlife watching) recreational groups [10]. Six sociodemographic attributes were used to infer whether different recreationists were participating in these three recreational activities; this information was used to gain further insight into potential participation and ecological effects. For instance, vehicle type can influence which areas recreationists can access [12]. We discuss our findings in the context of how a greater understanding of the social component of PPAs will aid decision making and lead to more informed and effective management actions, such as minimizing user conflicts, prioritizing conservation efforts, preserving ecological resources, and optimizing diverse recreational opportunities to support a growing number of recreationists.

## Methods and materials

### Study system

**Fig 1:**
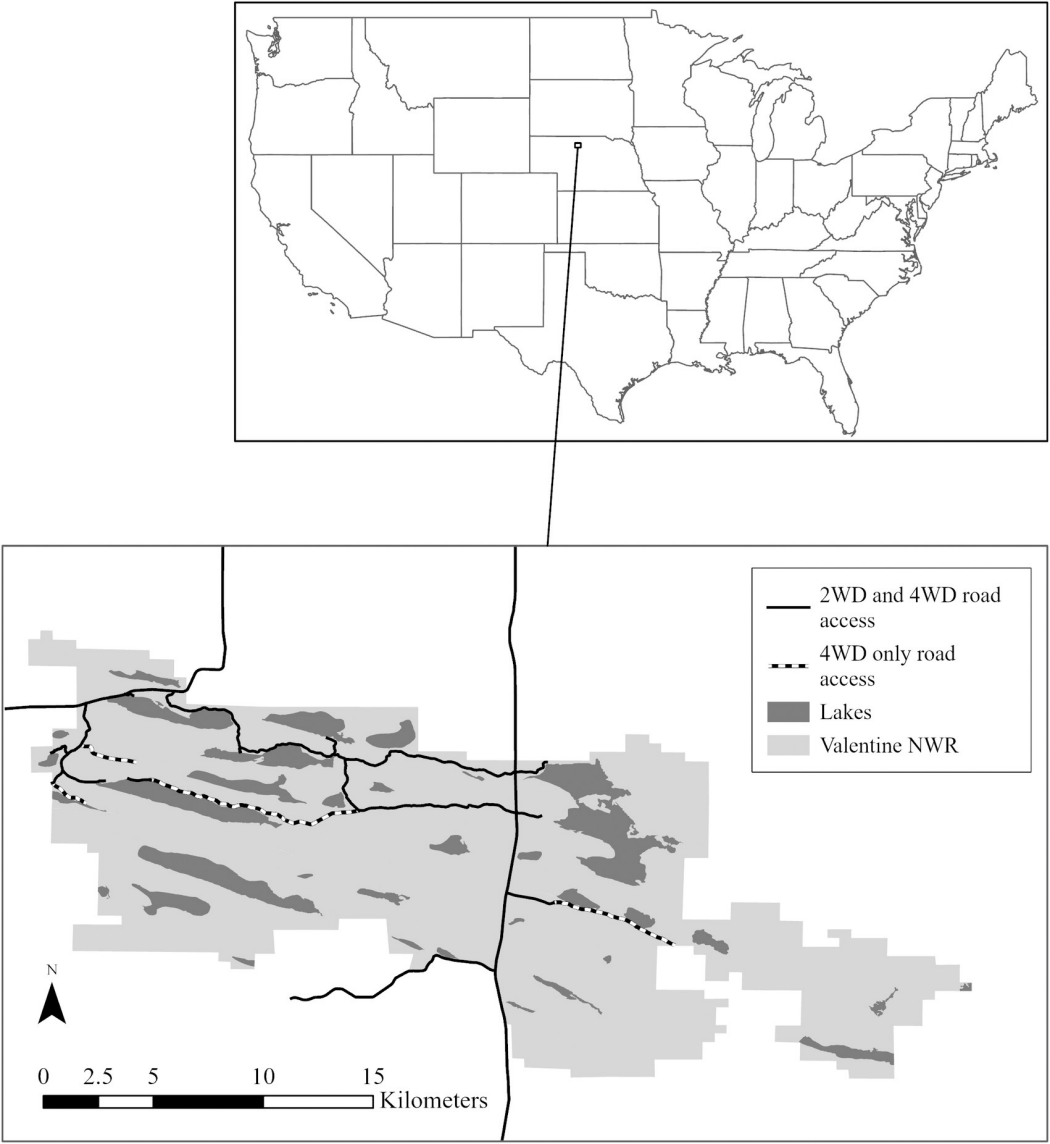
Map of Valentine National Wildlife Refuge in Cherry County, Nebraska, USA. Two-wheel drive (2WD) and four-wheel drive (4WD) road access is indicated on the refuge map.

The Valentine NWR is located in north-central Nebraska (Fig 1) and strives to balance preservation of 28,941 hectares of the Sandhills ecosystem while providing recreational opportunities for visitors. The Valentine NWR is also situated within a larger area that is predicted to generate high potential for developing ecotourism [13]. Refuge personnel determined recreational activities that were allowed on Valentine NWR based on compatibility with wildlife [10]. Allowed activities included a consumptive activity (hunting), an intermediate-consumptive activity (fishing), and non-consumptive activities (wildlife watching, touring, hiking, photography, and environmental education). Less common non-consumptive (i.e., other) activities included kayaking, rest stop, running, prospecting, ice checking, eclipse watching, and dog walking, but were not evaluated for compatibility in the Comprehensive Conservation Plan [10]. This refuge is closed to all recreationists from sunset to sunrise, and two Research Natural Areas within the refuge are closed to visitors.

### Surveys

Surveys were placed during one year (30 July 2017 to 26 July 2018) on the windshields of recreationists’ vehicles that were parked somewhere on Valentine NWR (S1 Survey). Surveys indicated in bold letters that participation was voluntary, and a link to a website was provided that contained information on participants rights along with contact information (mailing address, telephone number, and email address) for questions about the survey. Consent was implied by a participant answering questions and returning the survey. This study was approved by the University of Nebraska-Lincoln Institutional Review Board (Project ID 14051).

Distribution of surveys was stratified by two-week periods (fourteen days; [14]). Within each two-week period, days were further stratified by day type (weekday [Monday through Friday] and weekend [Saturday and Sunday]). Six weekdays and two weekend days were randomly sampled within two-week sampling periods. Each day was then stratified into either a morning or an evening sampling period. Morning sampling periods were initiated at sunrise and evening sampling periods were initiated eight hours prior to sunset (e.g., 11:00 start with a 19:00 sunset). Sampling routes were predefined; the start (and end) location and route direction (clockwise or counterclockwise) were randomized for each sampling day. Additional “event” days were added to the sampling schedule and included holidays and hunting openers. We expected deviations from normal use during these events and thus wanted to account for potential increased activity. We did not sample on foul-weather days (e.g., blizzards) and assumed no recreational activities occurred during these adverse weather events [15].

Respondents could select from seven permitted activities in which they participated that included fishing, hunting, wildlife watching, touring, hiking, photography, and environmental education or specify additional activities (i.e., other) that were not listed on the windshield survey. Respondents could return completed surveys in a drop box on refuge or through the U.S. Postal Service, prepaid and postmarked. Date, time, location, and vehicle type (two-wheel [2WD] or four-wheel [4WD] drive) for each survey distributed was recorded. Parties were subsequently categorized based on the consumptive hierarchical gradient of selected activities. For example, parties that selected hunting, regardless of other activities (e.g., hunting and hiking), were assigned to the consumptive group. Remaining parties that selected fishing, regardless of other activities (e.g., fishing and wildlife watching), were assigned to the intermediate-consumptive group. Remaining parties that selected wildlife watching, touring, hiking, photography, environmental education, or other activities were assigned to the non-consumptive group.

Sociodemographic information for the three groups was collected using information from returned surveys and included party size (individuals that travel and recreate together), senior (≥ 65 years) present, distance traveled (based on ZIP code), average household income, population type (urban or rural residence, also based on ZIP code), and vehicle type. Sociodemographics were used to describe groups and to understand whether the same or different recreationists were participating in various activities. Sociodemographics, such as age, income, and population type, can influence participation in certain recreational activities [16]. Understanding sociodemographics of recreationists can help minimize social and ecological problems, such as large party sizes (may characterize certain recreational-activity groups) that cause crowding or disturb wildlife [17]. Furthermore, different sociodemographic attributes among groups would suggest a heterogeneous group of recreationists (i.e., the same recreationists do not participate in different recreational activities throughout the year).

### Analysis

A Kolmogorov-Smirnov 2-sample test was used to compare temporal (two-week survey periods) distributions between the number of distributed surveys and returned surveys (i.e., respondents) to evaluate temporal non-response bias of recreational activities [18]. We would expect seasonal differences in response rates if certain groups (i.e., anglers during winter vs. hunters during fall) were less likely to respond to our survey [14, 19]. We identified a similar temporal distribution among the two-week survey periods between respondents and non-respondents (Kolmogorov-Smirnov test: D = 0.26, p > 0.32). Thus, the proportion of respondents did not significantly fluctuate among the two-week survey periods throughout the year, even though the types of recreational activities did fluctuate, with most consumptive use occurring in the fall and most intermediate-consumptive use occurring in the winter [14].

Frequencies of activities were calculated by summing returned surveys by activity group. Descriptive statistics were used to summarize sociodemographic attributes associated with each group. Distance traveled was calculated from refuge headquarters to center point of recreationist’s home ZIP code using ‘distHaversine’ function in R geosphere package [20]. We used ZIP code to categorize each party by population type according to the U.S. Census Bureau (urban ≥ 2,590 people per square kilometer [ppskm] or rural < 2,590 ppskm; [21]), and to determine average household income using Esri 2018 demographics database [22].

We used one-way permutational multivariate analysis of variance (PERMANOVA) to evaluate differences in sociodemographics among the three groups. The ‘adonis2’ function in the vegan R package was used to conduct PERMANOVA with 999 permutations [23, 24]. The PERMANOVA is robust, handling several variables together, including both continuous and categorical data [25]. Continuous attributes, which included party size, distance traveled, and average household income, were scaled using:

$$x^{\prime }=\frac{x-x_{min}}{x_{max}-x_{min}}$$

where x is the attribute value and *x*′ is the normalized value. After a significant PERMANOVA result, post-hoc pairwise comparisons were conducted using ‘pairwise.perm.manova’ function in the vegan package to determine differences in group mean dispersions. We conducted *a posteriori* univariate comparison for each attribute to understand attributes contributing to a significant PERMANOVA result. We tested the assumption of homogeneity of multivariate dispersion between groups (consumptive vs intermediate-consumptive, consumptive vs non-consumptive, and intermediate-consumptive vs non-consumptive) using the ‘betadisper’ function in vegan package.

## Results

Of the 2,251 surveys distributed, 861 were returned (38% return rate). Of the 861 returned surveys, 789 completed all necessary questions (35% functional return rate) and were used for subsequent analysis, with all recreational-activity groups present on this refuge. We discovered that the intermediate-consumptive group was the most dominant with 616 (78%) parties representing this group, followed by 95 (12%) parties representing the consumptive group, and 78 (10%) parties representing the non-consumptive group.

The intermediate-consumptive group had the greatest rank order for party size and traveling in 4WD vehicles (mean party size = 3; 4WD = 96%), followed by the consumptive group (mean party size = 2; 4WD = 94%) and the non-consumptive group (mean party size = 2; 4WD = 72%). The non-consumptive group had the greatest rank order for seniors present and residing in urban areas (seniors present = 44%; urban = 31%), followed by the intermediate-consumptive group (seniors present = 31%; urban = 14%) and the consumptive group (seniors present = 28%; urban = 11%). The non-consumptive group also had the greatest rank order for average distance travelled and household income (mean distance travelled = 863 km; mean income = $83,695), followed by the consumptive group (mean distance travelled = 818 km; mean income = $78,968) and the intermediate-consumptive group (mean distance travelled = 260 km; mean income = $70,253).

**Fig 2:**
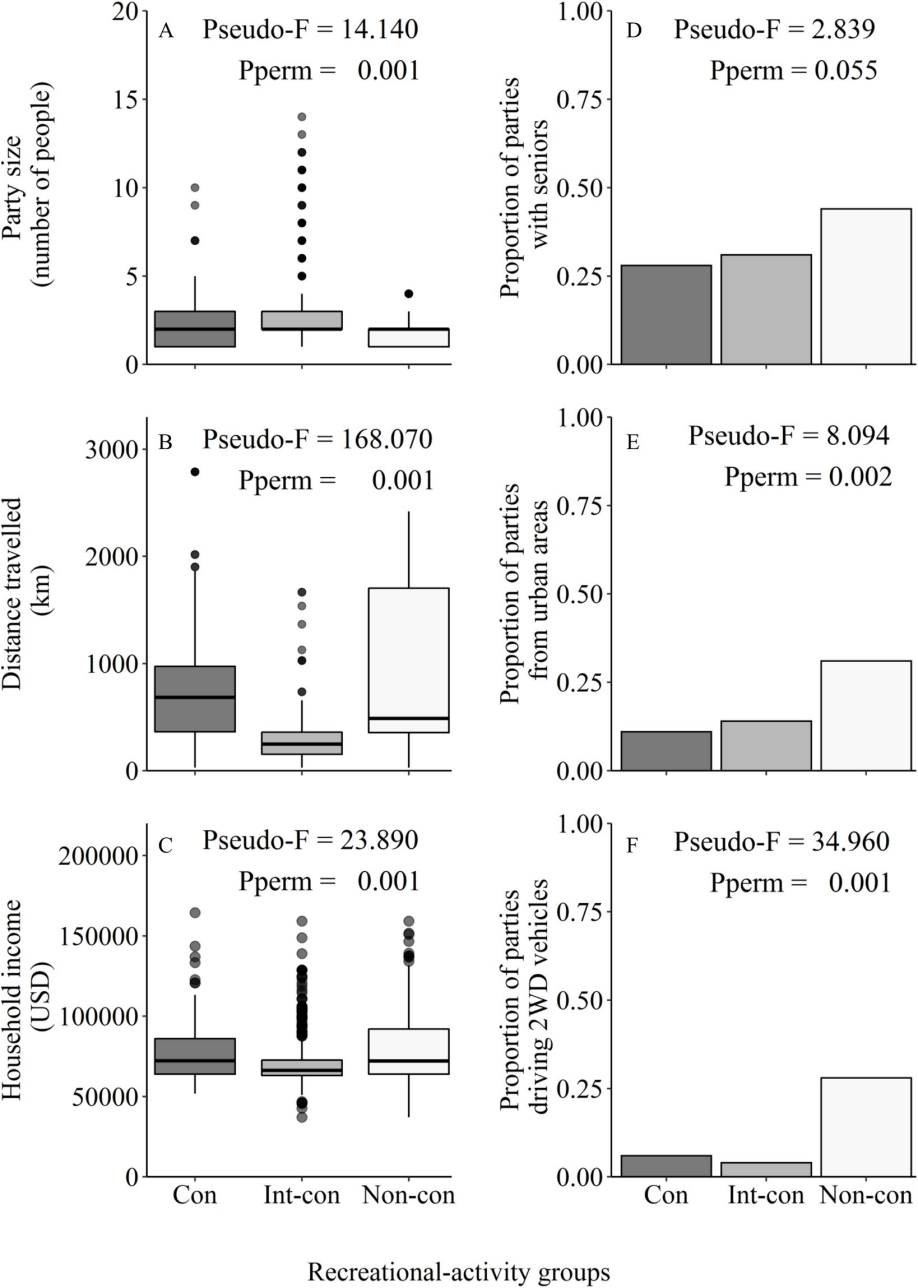
Box plots and bar graphs of the sociodemographic attributes of the consumptive (Con [hunting]; dark gray), intermediate-consumptive (Int-con [fishing]; medium gray), and non-consumptive (Non-con [e.g., wildlife watching]; light gray) recreational-activity groups surveyed at Valentine National Wildlife Refuge during 2017–2018. Box plots (A-C) illustrate attribute variability for party size, distance travelled, and household income among surveyed groups. Horizontal lines represent the median, boxes represent the range from 25th to 75th percentile, upper whiskers extend from box to largest value at most 1.5 * IQR (interquartile range), lower whiskers extend to lowest value no further than 1.5 *IQR, and points represent outliers. Bar graphs (D-F) illustrate proportions of surveyed parties with seniors present (≥ 65 years), from urban areas (≥ 2590 people per square kilometer) and driving two-wheel drive (2WD) vehicles for surveyed groups. Univariate results of PERMANOVA examining attribute variation among groups are indicated on each plot (Pseudo-F value by permutation; p-values based on 999 permutations [Pperm]), and all attributes had degrees of freedom of 2.

Sociodemographic attributes varied across the three groups (Pseudo-F = 15.961, df = 2, P_perm_ = 0.001; Fig 2); pairwise comparisons revealed all recreational-activity groups were significantly different from each other (P_perm_ < 0.001). Post-hoc univariate PERMANOVA revealed significant differences among the three groups for each attribute, except the senior attribute. Analysis of homogeneity of multivariate dispersion between groups was significant. There was greater dispersion in attributes among the non-consumptive group compared to the consumptive and intermediate-consumptive groups. Although PERMANOVA tests are susceptible to differences in dispersion, we interpret our findings to indicate that sociodemographic attributes varied both within and across the three groups [26]. Thus, there were three distinct and diverse recreational-activity groups, consisting of recreationists with different sociodemographics.

## Discussion

Valentine NWR supports heterogeneous groups of recreationists that participate in consumptive, intermediate-consumptive, and non-consumptive activities. Based on the significant sociodemographic differences among groups, we presume that different recreationists were participating in different activity types. Recreationists differed across most attributes, including party size, distance traveled, household income, population type, and vehicle type. These sociodemographic differences among recreational-activity groups have important implications for management of Valentine NWR and the ability to support diverse recreational-activity groups. Some groups may have more recreational opportunities than others, which could vary across segments of society [27, 28]. For instance, non-consumptive users were more likely to drive 2WD vehicles and may have limited access within and across different PPAs. Thus, management efforts to increase wildlife viewing or other nonconsumptive activities could consider providing more 2WD accessible roads. Although diverse, we identified that Valentine NWR primarily supports the intermediate-consumptive group. Overlooking recreational diversity and the predominance of one recreational-activity group could be problematic when allocating resources and implementing different management actions. For instance, catering to the predominant recreational-activity group (e.g., anglers) by providing greater access and subsequent use in certain areas (e.g., larger parking lots, longer fishing docks, and trails around the lake and lakes open to fishing) could attract and concentrate diverse users, such as bird watchers and anglers using a dock or shoreline trails and lead to congestion, elevated social conflicts, and deleterious ecological impacts [29]. Although it will require resources, it is important to identify and manage for these heterogeneous activities and recreationists to achieve the dual goals of PPAs.

The current participation and sociodemographic information suggests that certain groups may be limited in their opportunity to recreate at Valentine NWR. Monetary constraints could limit access to (e.g., long-distance travel) and within (e.g., 4WD vs. 2WD vehicles) the refuge [27, 30–32]. The consumptive group traveled a greater distance to reach Valentine NWR, had a higher income, and a greater proportion resided in rural areas than the intermediate-consumptive group. Although surrounding areas of Valentine NWR are rural, the unique ecosystem and recreational opportunities at the NWR appeared to attract many consumptive recreationists with higher incomes from farther distances. Some consumptive recreationists may be travelling from a different state, thus refuge managers may want to include hunting regulations and maps at kiosks for those unfamiliar with Nebraska’s game regulations. The non-consumptive group was least represented on the Valentine NWR, which could be a result of limited opportunities compared to other groups. Non-consumptive recreationists had a greater proportion of urban residents and drove more 2WD vehicles than the consumptive and intermediate-consumptive groups. With no urban areas near the refuge (> 210 km), non-consumptive recreationists had to travel a greater distance and expend more money than the consumptive and intermediate-consumptive groups to visit Valentine NWR. The unique landscape and wildlife of this region may be attracting non-consumptive recreationists and thus providing important ecotourism to the refuge and nearby rural communities. Knowledge of sociodemographics associated with different groups can allow managers to better understand potential limitations to participation and how this may affect ecotourism.

We acknowledge that our study only focused on a single PPA; however, we anticipate that most PPA’s that allow both nonconsumptive and consumptive recreational activities could benefit by understanding the frequency and sociodemographic attributes of these recreational activity groups. Recognizing differences in frequency and sociodemographics among groups can aid in management decisions to accommodate greater or fewer recreationists, depending on their relative ecological impacts [5]. Some birds have a reduced tolerance for large recreating parties [17] and recreationists that go off-trail can impact sensitive flora and fauna [5] such as disturbing nesting grassland birds or trampling the endangered Blowout Penstemon at Valentine NWR. Consequently, managers may want to limit access to areas with sensitive species to minimize recreation impacts [33]. This could be as simple as limiting 2WD accessible roads in areas where at-risk birds breed that would attract non-consumptive users if they had access. Managers may also want to create trails to limit off-road dispersal [34] and smaller parking areas and spatially separated recreational opportunities, such as providing non-consumptive activities along paved roads away from lakes open to fishing, to ease crowding and social conflicts among different groups [35]. Tracking spatial and temporal patterns of each recreational-activity group could expose potential hotspots or areas with high recreational intensities [14]. We contend that a better understanding of the diversity of recreationists at PPAs would allow managers to make more informed management decisions [4, 35].

Many PPAs, including Valentine NWR, offer diverse recreational activities. Identifying heterogeneity among recreational-activity groups is essential to provide a multi-faceted management regime that fulfils the dual goals of preserving ecological systems and providing recreational opportunities. These dual goals may be viewed as competing goals by PPA managers as they face an increase in visitation. PPAs continue to face a decline in resources, thus making management of these valuable areas even more difficult [36]. Recognizing and accounting for diverse recreationists and activities will afford managers of PPAs the ability to concomitantly manage for diverse recreational-activity groups, prioritize conservation efforts, and preserve ecological resources.

## Supporting information

S1 Survey (PDF)
