# Supplementary material for: Heterogeneity of recreationists in a park and protected area
Source: PLoS One. 2022 May 11;17(5):e0268303. doi: 10.1371/journal.pone.0268303 (PMC9094530; doi:10.1371/journal.pone.0268303)
Supplement: S1 Survey — (PDF) [file pone.0268303.s001.pdf]

VALENTINE NATIONAL WILDLIFE REFUGE
RECREATIONAL EXPERIENCE SURVEY

NEBRASKA COOPERATIVE FISH & WILDLIFE RESEARCH UNIT

Hello! We left this **voluntary** research survey on ____/____/____ at ____:____. Your participation will help us understand use of Valentine National Wildlife Refuge. Participant information is available at *http://FishHunt.unl.edu*.

PLEASE TELL US ABOUT YOUR VISIT TODAY.

Your responses on **today's activities** are valued, *even if you completed this survey on a **prior** visit*.

INFORMATION ABOUT YOUR GROUP

❶ **Recreational activities** today at this refuge (*check all that apply*)

☐ Fishing ☐ Hiking ☐ Wildlife Watching ☐ Other__________________
☐ Hunting ☐ Touring ☐ Photography ☐ Environmental Education

❷ **Refuge lakes** visited today? (*check all that apply*)

☐ No lake visited

open to waterfowl hunting and fishing: ☐ Duck ☐ Rice ☐ Watts

open to fishing: ☐ Clear ☐ Dewey ☐ Hackberry ☐ Pelican ☐ West Long ☐ Willow

☐ Baker	☐ Devils Punch Bowl	☐ Lee	☐ North Marsh
☐ Center	☐ East Long	☐ Little Hay	☐ Middle Marsh
☐ Coleman	☐ East Sweetwater	☐ Lost	☐ South Marsh
☐ Cow	☐ West Sweetwater	☐ McKeel	☐ School
☐ Crooked	☐ East Twin	☐ Mule	☐ Tom's
☐ Dads	☐ West Twin	☐ Pony	☐ Whitewater

❸ Number of **people** in your group (*1 = you alone*)? ________

❹ **Number** of group members: 17 years or younger? _______ 18-64 years? _______ 65 years or older? _______

❺ **Zip codes** of home residences for group members? ___________ ___________ ___________ ___________

INFORMATION ABOUT FISHING, HUNTING AND WILDLIFE WATCHING

❻ If you **fished** on this refuge today:

Lake Name:	1.____________		2.____________		3.____________	
Time fished:	___hrs	___mins	___hrs	___mins	___hrs	___mins
Number of people:	_______		_______		_______	
Number of fish:	Released	Kept	Released	Kept	Released	Kept
Black crappie	_____	_____	_____	_____	_____	_____
Bluegill	_____	_____	_____	_____	_____	_____
Largemouth bass	_____	_____	_____	_____	_____	_____
Northern pike	_____	_____	_____	_____	_____	_____
Yellow perch	_____	_____	_____	_____	_____	_____
Common carp	_____	_____	_____	_____	_____	_____
Other ___________	_____	_____	_____	_____	_____	_____

❼ If you **hunted** on this refuge today:

Time hunted: ____hrs ____mins
Number of people: ______
Number of animals harvested?
(*0 = hunted but none harvested; NA = did not hunt*)

Waterfowl ______
Prairie grouse ______
Pheasants ______
Dove ______
Deer ______
Coyote ______

❽ If you **watched wildlife** on this refuge today: Time spent: ____ hrs ____mins Number of people: _____

Rank, in order of preference, the groups of animals that you watched? (1 = *most preferred; NA = did not watch*)

Insects ______ Fish _____ Amphibians _____ Reptiles _____ Birds ______ Mammals ______

COMMENTS

PLACE COMPLETED SURVEY IN DESIGNATED BOX — look for gold star ★ — **OR IN THE U.S. MAIL.**

Thank you for completing this survey! Your responses will help improve management of our natural resources.
